# A critical discourse analysis of Trump's election campaign speeches

**DOI:** 10.1016/j.heliyon.2022.e09256

**Published:** 2022-04-06

**Authors:** Enas Naji Kadim

**Affiliations:** Wasit University, Wasit, Iraq

**Keywords:** Discourse, Discrimination, Ideology, Power

## Abstract

This article presents a study in critical discourse analysis of Trump's election campaign speeches. Trump's speeches against immigrants have raised controversy in the world in general and in the United States in particular. Such speeches against immigrants might motivate Native Americans to enter a racial conflict with Muslims and other non-native Americans. The study adopts Van Dijk's (1991, 1995; 2006) discourse strategies. Therefore, this study aims to show how Trump ideologically invested these discursive strategies in the positive discourse of US (Trump, the majority of American people) and the negative discourse of THEM (Obama, Obama administration, Clinton, immigrants, especially Muslims). Based on data analysis, the study found out that Trump ideologically invested the (12) discourse strategies in almost all of the selected speeches for political interests. The study is hoped to encourage politicians to stop using language that is full of racism and sectarianism, because such language has negative results on innocent civilians.

## 1 Introduction

Richards and Schmidt (2010) define CDA as a form of discourse analysis that takes a critical stance towards the way language is used. CDA aims to critically analyse texts and other types of discourse in order to uncover hidden ideologies, domination, manipulation, power, and racism underlying them.

Fairclough (1995) states that:

By CDA I mean discourse analysis which aims to systematically explore often opaque relationships of causality and determination between (a) discursive practices, events and texts, and (b) wider social and cultural structures, relations, and processes: to investigate how such practices, events and texts arise out of and are ideologically shaped by relations of power and struggles over power, and to explore how the opacity of these relationships between discourse and society is itself a factor securing power and hegemony (132).

Widdowson (2007) illustrates that CDA is concerned (with and about) the use and abuse of language for ideological and powerful purposes. In this case, critical discourse analysts set themselves to discover and trace theses ideologies in texts that are used by powerful groups and institutions. Wodak and Meyer (2001) demonstrate that CDA is an analytical framework that is used to extract opacity and make everything clear to people. CDA is also used to illustrate and analyze notions such as power, dominance, control, and ideology in language. Trask (2007) emphasises that CDA is basically interested in the social context in which a text is written. It is easy for CDA to analyze texts through the vocabulary and structures (the linguistic devices that are used to relate one part to another). CDA is concerned with social problems and global sensitive issues and the role of discourse in the production and reproduction of power abuse, domination, ideologies, inequalities, and injustice as manifested in language (Van Dijk, 2001a).

Trump's speeches show opposition to Islam and other immigrants. This opposition and hostility will absolutely agitate Native Americans against other American citizens who came from other countries. I select different discourse strategies from Van Dijk's (1991, 1995; 2006). The selected (12) discourse strategies can be invested ideologically by politicians and media. Politicians resort to these strategies for persuasive purposes. In other words, they push people to believe in their own ideologies. Therefore, this study aims to show how Trump ideologically invested these discursive strategies in the negative representation of Obama, Obama administration, Clinton, and immigrants especially Muslims on one hand, and the positive representation of Trump, Native Americans, Christians, and Europeans on the other.

## 2 Literature review

Tehrani and Yeganeh (1999) point out that CDA aims to uncover the insinuation of ideology and imposition of power into texts which ordinary people cannot notice.

CDA is a type of analytical framework that is concerned with analysing and clarifying issues such as power, inequality, control, and dominance in texts and talk (Van Dijk, 2001b). The job of critical discourse analysts is to extract hidden ideologies from texts and make them clear to people (Fowler, 1991; Fairclough, 1993; Batstone, 1995).

According to Trask (2007), CDA is interested in answering questions such as why was this text constructed at all? To what people or societies is it addressed? and why? Does the writer or speaker have concealed purposes? What hidden assumptions and biases underlie the text?

Joseph (2006) illustrates that politicians and media owners use propaganda, deception, and manipulation in their speeches to achieve political goals and interests on one hand, and to diminish the value or reputation of their opponents on the other.

Text producers, especially politicians use language in a particular way through using many different manipulative strategies to achieve their objectives. CDA aims to discover the real intentions of text-producers to lay people. In this case, it would be important to identify the manipulative and implicative techniques that are used by text-producers to persuade people (Van Dijk, 1997).

In actual fact, Wilson (1990) concludes that language of politicians not only conveys the message to the public but also it is not free from manipulation, deception, and persuasion.

Van Dijk (2000) illustrates that ideology plays a crucial role in the positive representation of certain groups and the negative representation of others. The discourse of *SELF* (positive and good) and *OTHERS* (negative and bad) is always used by politicians and media. In other words, the in-group members are always represented in a positive way, while the out-group members are always represented in a negative way:

• Say positive things about US.
• Say negative things about THEM.
• Do not say negative things about US.
• Do not say positive things about THEM.

Ideology is defined as a set of beliefs or ideas shared by certain groups and institutions about a specific topic. According to Shelby (2003), philosophers and social theorists list a set of properties that characterize ideology as follows:

1. Ideology is a set of beliefs that change certain false assumptions, about crucial and important matters, which have direct influence on many groups and societies, into true to achieve manipulative and persuasive purposes.
2. Ideology functions as “meaning in the service of power”. In other words, certain groups use them to achieve power, domination, and control over others.
3. Ideology is used to legitimate the interests of the powerful groups in certain societies.
4. Ideology is a set of beliefs and rules that can manipulate and persuade people for the interests of the dominated group in society.
5. Ideology is distorted assumptions that can be used to conceal social contradictions for the sake and interests of the dominant societies.
6. Ideology is generally distortive and reflects powers of domination.

Luke (1974) illustrates that Y uses power over Z by forcing Z to do what he/she wants him to do. But Y also uses other forms of power to control Z thoughts and desires such as manipulation, brainwashing, deception, and propaganda. Politicians and media owners know that such forms of power and control can only be achieved through the skilful use of language.

## 3 Method

This section is concerned with the selection and description of the research data, Trump's life and his election campaign on one hand and the analytical framework (tools of analysis on the other.

### 3.1 Data

The data used in the analysis are made up of (30) selected quotations taken from Trump's political speeches on Obama, Clinton, and Immigration. I focus on certain quotations which concern Trump's views on Obama, Clinton, terrorism, Muslims, and immigration. The data are taken from American media websites and Trump's websites.

Donald Trump is the 45^th^ president of the United States of America. Trump was born in Queens, New York City on the 14^th^ of June, 1946. His father, Frederick Trump was a builder and a wealthy real estate developer. His mother is Mary Anne Macleod Trump. Trump's parents sent him to Military Academy at age 13. At the Academy, Trump did well on both: the social and academic sides becoming a star athlete and student leader by the time he graduated in 1964 (https://www.biography.com/us-president/donald-trump).

Trump, then graduated from the University of Pennsylvania in economics, 1968. After his graduation, he became a successful businessman. He built lots of hotels, casinos, and office towers. This allows him to collect a net worth of billions and fame on television. Trump wanted to expand his ambitions in politics. Therefore, he entered national politics and he was active from the 1980s until present time. Trump expressed his wishes to run for presidency as a republican from 2004 to 2012. In June 2015, he announced that he would be a candidate for presidency of the 2016 US election campaign. Trump's election campaign was characterized by opposition to immigration, Islamic terrorism, Obama and Hilary Clinton administration. His election campaign was also characterized by supporting American jobs, trade, and economy (https://www.thefamouspeople.com/profiles/donald-trump-3378.php).

### 3.2 Tools of analysis

Van Dijk's (1991, 1995; 2006) frameworks of analysis focus on the micro levels of political discourse, that is the meaning, style, and rhetoric of its lexical items and sentences. According to Van Dijk (1991, 1995; 2006), there are a number of discourse strategies of the local meaning of political discourse. These discourse strategies are summarized as follows:

1. Implication

Implication is a strategy used by media owners, journalists and politicians to communicate an idea or feeling without saying it directly. People imply something by what they say. But the listener or receiver infers something from what somebody else says (Van Dijk, 1991).

2. Presupposition

According to Yule (2010), a presupposition is an assumption by a speaker/writer about what is true or already known by the listener/reader.

3. Hyperbole

Politicians use hyperbole to exaggerate. They deliberately do this to emphasize something, to add humour, to gain attention, interests, or achieve political aim (Van Dijk, 1995; Abbas, 2019; Cruse, 2006).

4. Compassion Move

This strategy shows sympathy for (the weak) or innocent civilians by the wrong policies or actions of others to achieve political interests (Van Dijk, 1995).

5. Negative Comparison

This type of strategy is used to emphasize the bad actions and qualities of others to achieve key gains and interests (Van Dijk, 1995).

6. Blaming the Victim

Politicians make use of this strategy through reversing the blame by attributing it to the opponent (Van Dijk, 1991).

7. Contrast and Division

This strategy is used by politicians against their opponents. It is often found in election campaigns. Politicians always present themselves as "good", while their opponents are "bad" (Van Dijk, 1991).

8. Actor Description

Actor description means the way people ideologically describe certain actors or participants. The in-group members are always good and positive, while the out-group members are always described as bad and negative (Van Dijk, 2006).

9. Number game

Politicians resort to use numbers and statistics in order to persuasively achieve credibility and objectivity (Van Dijk, 2006).

10. Metaphor

Metaphor is a powerful ideological tool that can be used for negative representation of certain groups and positive representation of others (Van Dijk, 2006).

11. Repetition

In media and political discourse, repetition can serve as a powerful ideological strategy to positively represent the in-group members and negatively represent the out-group members (Van Dijk, 2006).

12. Pronouns

The power of pronouns can be invested ideologically by politicians. Pronouns such as I, We, They are powerful and ideological. The pronoun ‘I’ is used to show one's good qualities and achievements, ‘We’ is used to show unity and solidarity, while ‘They’ is used to show others' bad and negative qualities.

## 4 Data analysis

In this section, I present the analysis of (30) quotations taken from Trump's political speeches on American foreign policy, Terrorism, Islam, and Immigration during his election campaign for U.S. Presidency. Quotations are chosen from Trump's speeches on American foreign policy. The focus will be on Trump's controversial views on Islam, immigration, Obama administration, and his opponent from the Democratic Party, Hilary Clinton. The selected quotations are listed in the appendix section at the end of the article.

In quotation (1), the verb "hate" means 'a very strong feeling of dislike; intense hostility usually deriving from fear or anger'. Trump's use of the general term "Islam" refers to his ignorance of this peaceful religion and its innocent people. The statement "Islam hates us" presupposes that all Islam (religion and people) hates American people without even drawing little distinction between this peaceful religion and its people and some terrorist organizations which have nothing to do with Islam. The adjective "tremendous" means 'very large or great; being such as may excite trembling or arouse dread, awe, or terror'. The adjective "tremendous" illustrates the deliberate exaggeration used by Trump to manipulate the public and motivate the American society against Muslim minority in the United States. This exaggeration is reinforced when Trump mentions the phrase "can't allow". The modal "can" with the negative marker "not" is used to refuse permission. It is similar to prevention in impolite way because "can" is considered rude in grammar (Swan, 2005). The phrases "can't allow" and "not Muslims" illustrate the positive and good image of Trump and the majority of American people, while the negative and bad image is represented by Muslims and other immigrants in the United States. The theme of "division and contrast" is clear in this quotation. The general phrase "it is hard to define" demonstrates Trump's insistence that all this terror comes from Islam.

In all of the (2–7) Trump's quotations, discourse strategies such as implication, presupposition, and hyperbole are used clearly for ideological purposes. The themes of division and contrast, blaming the victim, compassion move, and negative comparison are presented by Trump in more manipulative and persuasive ways to achieve interests at the expense of Islam, Muslims, and other immigrants.

There is a clear tendency from Trump to put the blame of all the terrorist incidents in the United States and other European countries such as France, Belgium, and Germany on Muslim people.

Trump deliberately exaggerates too much when mentioning the phrases "*It was the worst mass shooting in our history, and the worst attack on the LGTBQ community in our history*". This is too much exaggeration and also not true, because the American history is full of "mass shootings" especially during the American civil war and Black-White conflicts. The acronym "LGTBQ" stands for 'Lesbian, Gay, Transgender, Bisexual, Queer or Questioning'. The "LGTBQ" community is forbidden in the Islamic religion and those who practise it are considered disobedient. The use of this acronym implies that Trump wants to say that those who commit this crime at the Pulse Nightclub in Orlando are Muslims because Muslims consider these types of people as disobedient and they should be banned.

In quotation (4), Trump mentions the prophet Mohammed. The prophet Mohammed is the messenger of God and the leader of the Islamic religion and nation. Trump presupposes that the terrorists who attacked the French and Jewish newspapers which made fun of the prophet Mohammed are Muslims.

In quotation (6), Trump becomes so clear when mentioning the phrase "an Islamic terrorist". According to Trump, the terrorist who killed tens of innocent people is simply a Muslim without even drawing distinction between Islam and the so-called "radical Islam". In quotation (7), the "refugee" who killed some German people according to Trump is coming from Islamic countries, because it is known to everybody that Germany is the first country which opened the borders to Muslim immigrants.

The word "extermination" in quotation (7) means 'to destroy or kill a group of people completely'. It involves too much exaggeration by Trump, because not only Christians have suffered from ISIS crimes but also other different religions and sects such as Yazidis, Kurds, Alawites, Druzes, Sunnis, and Shia.

In terms of compassion move, Trump always shows signs of sympathy and solidarity for the majority group of Americans in particular against the violent actions of Muslims. The images of negative comparison and division and contrast are clear in Trump's speeches. The discourse of "US" and "THEM" is clear in the aforementioned quotations. The majority group of Americans is presented as good, peaceful, and innocent people, while Muslims are presented as bad, terrorists, and evil people. The Muslims are always associated with violence. The image of blaming the victim is also clear in the quotations. In all of the analysed quotations, the victim is Islamic religion and Muslims. According to Trump, Islam and Muslims are to blame and they are behind the violence and crimes in Europe and the United States.

In the (8–15) quotations, Trump uses negative comparison, division and contrast, and compassion move clearly and in a more manipulative and persuasive way. Trump started by criticizing Obama, Clinton, and their foreign policy administration. He describes the Obama administration as having no vision, no purpose, no direction, and no strategy. According to Trump, the Obama administration has weakened the military, economy, overextension, betrayal of friends (Israel), while flirting with the enemy (Iran). Obama administration also according to Trump lacks respect focusing on two main events happened with Obama in Cuba and Saudi Arabia. Trump also criticizes Obama and Clinton for refusing to call the terrorists as he calls them "radical Islam" (*And we're in a war against radical Islam, but President Obama won't even name the enemy! Hillary Clinton also refuses to say the words "radical Islam")*. The word "even" is used to stress something that is surprising or unlikely; it is also used to stress the difference between two things that are being compared. The lexical item "even" is used to show a sense of disagreement between him and his opponents from the Democratic Party. Trump uses the modal "must" in quotation (12) which is used to say what is necessary and to give strong advice and orders to other people. According to Trump, Islam generates extremism and terrorism; therefore, Muslims should be banned from entering the United States. In quotation (14), the conjunction "and" implies the concepts 'in addition' or 'plus'. It is used as a function word to indicate connection or addition, especially of items within the same class or type: Trump wants to say that both Obama and Clinton are the same and they both have made the American foreign policy reckless, rudderless, and aimless. The statement "*blazed a path of destruction in its wake*" refers to the wrong policies of Obama and his follower Clinton. It involves too much exaggeration from Trump.

The statement "*I am the only person running for the Presidency who understands this problem and knows how to fix it*" presupposes that the Obama administration, Trump's rival from the Democratic Party, Hillary Clinton, and all Trump's opponents cannot solve the U.S. problems. The only person who can solve these problems is Trump. The other statement "*Iran cannot be allowed to have a nuclear weapon and, under a Trump administration, will never be allowed to have a nuclear weapon*" also presupposes that Iran, under Obama administration and Clinton, has been allowed to have nuclear weapons through the "disastrous deal" with the United States. The statement *"Israel, our great friend and the one true democracy in the Middle East, has been snubbed and criticized by an administration that lacks moral clarity*" also presupposes that under Trump administration, Israel will be treated better than Obama's administration and it will be treated better than other U.S. allies in the region. It also presupposes that all regional countries have dictatorship administration except Israel. The statement "*Under a Trump administration, no American citizen will ever again feel that their needs come second to the citizens of foreign countries*" presupposes that under Obama administration, Native Americans have felt that their rights and needs come second to other non-native Americans (immigrants).

In most of his speeches, Trump often shows sympathy, compassion, and feeling of support for the majority group of Americans in particular. The majority group of Americans are always presented as affected, influenced by, and victims to the Islamic terrorism. Most of Trump's speeches are full of compassion move. Compassion move is a strategy used by Trump to achieve political goals. Trump often manipulates the public by mentioning the wrong policies of his predecessor's administration and his opponent, Hillary Clinton. Quotation (11) is the best example of this strategy. In this quotation, Trump mentions the statements "*We left Christians subject to intense persecution and even genocide*". He shows sympathy for the Christians forgetting all the genocides and persecutions that happened to Muslims and other minorities in Syria, Iraq, and Libya due to terrorism. In the statement *"After Secretary Clinton's failed intervention in Libya, Islamic terrorists in Benghazi took down our consulate and killed our ambassador and three brave Americans. Then, instead of taking charge that night, Hillary Clinton decided to go home and sleep! Incredible"*, Trump becomes so manipulative in a way to motivate the public against Islam and his opponent Clinton. Trump clearly mentions the phrase "Islamic terrorists" killing American Ambassador and three other brave Americans. The adjective "incredible" means 'difficult or impossible to believe'. It motivates people to take negative impression towards Clinton.

The discourse of "US" (selves) and "THEM" (others) is clearly presented in most of Trump's speeches. The image of "US" (the good, innocent, and peaceful) is represented by (Trump, and the majority of Americans) who suffered from the wrong policies of Obama administration and Clinton's mistakes on one hand and Islamic terrorism on the other. The image of "THEM" (the bad and terrorist) is presented by (Obama, Clinton, Muslims, and other immigrants).

In the (16–21) quotations, the discourse of selves and others is clearly used by Trump. Starting with Quotation (16), the pronoun ‘they’ is used to show the bad and negative qualities of Mexicans. The pronoun ‘you’ in the sentence “they're not sending you” is used to refer to the Americans that Trump is addressing. It shows comparison between the Mexicans and the Americans. In other words, the Mexicans are bad and negative, while Americans are good and positive. The repetition of ‘they're’ and ‘they're bringing’ is used to show the negative qualities that the Mexicans will bring. In terms of actor description, Mexicans are described and identified as rapists bringing problems, drugs, and crimes. In quotation (17), Trump describes himself as a powerful actor that will bring America's greatness back. He uses sport metaphor (*I will be America's greatest defender and most loyal champion*) and presents himself as a loyal player that will defend America's goal and keep it clean. He will also score many goals leading America to victory. The pronoun ‘we’ is used deliberately by Trump. It shows unity and solidarity between the American people and him. He wants to say that all the American people including Trump himself have the same interests and goals. In quotation (18), Trump identified himself as the people's voice, the voice of the (*crying mothers who have lost their children because our politicians put their personal agendas before the national good*). The word “politicians” is used to refer to Obama and his administration. Division and contrast strategy is clearly used here. According to Trump, Obama and Clinton are bad and negative. Trump's language is sympathetic not only with the crying mothers who lost their children due to the wrong policies of the Obama and Clinton administration but also he is sympathetic with the jobless Americans in all of America due to the corrupt system and its wrong policies. Under Trump administration, all Americans will get jobs and all foreign employees will be fired.

In quotation 19, the word “change” is repeated (6) times. The repetition of the word “change” with the pronoun ‘we’ illustrates that Trump along with the American people want a new government, a government from the people and to the people. The word “rigged” is associated with false, fake, bad, rotten, deceitful, disloyal, and unfaithful. It is used to refer to the Obama administration which works for the insiders. The word “insiders” is also associated with deceitfulness and unfaithfulness. It is used to refer to Obama's administration. In quotation (20), the pronoun ‘she’ is used to refer to Hillary Clinton. In terms of actor description, Trump identified Clinton as a *dangerous liar*. The pronouns (us and we) are used to include Trump and the American people. Both are suffering from the wrong policies of Clinton. According to Trump, ‘economy is power’. This power has been destroyed and eliminated by Clinton's wrong policies. America is not the sole super-power anymore. This super-power cannot be brought back without a Trump administration. Quotation (21) is devoted to the immigrants who are immigrating to the United States and coming from what Trump calls “terrorist states”. The pronoun ‘our’ in the phrase “our country” is used to refer to the native Americans including Trump but excluding the immigrants and the politicians with their wrong policies of bringing more immigrants to the United States. The use of number game strategy flavoured with hyperbolic taste in the sentence “*We have thousands and thousands of people from certain terrorist states*” is used to plant fear in the hearts of the Native Americans regarding the risk that immigrants might bring. The pronoun ‘they’ is used to illustrate that those immigrants are bad and negative and they should not be included with the good and positive Native Americans. Immigrants are metaphorically compared to a Trojan horse. The idea of the Trojan horse was the cause behind the destruction of Troy. According to Trump, Obama and Clinton have been deceived by those immigrants (Trojan horses) who will burn and destroy the United States. Blaming the victim is another strategy used by Trump for political interests. Consider this sentence “*remember those horrible, disgusting people? “The Boston bombers arrived through the political asylum process*”. Obama and Clinton's wrong administration have brought criminals and terrorists that killed innocent Native Americans.

In the (22–30) quotations, Trump's inflammatory discourse is ideological. According to Trump, the terrorists that are killing and threatening the lives of the American and European people are Muslim immigrants. Trump mentions their origins and nationalities clearly. They are from Morocco, Uzbekistan, Syria, Somalia, Iraq, Pakistan, Yemen, and Afghanistan respectively. It should be noted that these countries have Muslim majority. It should also be noted that most of these countries are Arabic. In Trump's rhetoric, those Arabic-Muslims are associated with violence. This violence is directed towards Americans and Europeans. According to Trump, Muslim terrorists are busy, killing and wounding innocent people, blowing up buildings, building massive and very dangerous bombs, and pledging allegiance to and joining ISIS. Powerful and hyperbolic negative lexical items are used widely in Trump's rhetoric, especially when he talks about immigrants, Islamic terrorism, and Obama and Clinton. Words such as *massive, massively, very, dangerous, execution, bombs, bombers, shooting, slaughtering, gravely, and mass murder* are used to describe Muslim immigrants. All of these negative words are used to Muslims' negative acts in America and Europe. In quotations (27) and (28), the terrorist attacks are presented ideologically by Trumps. Trump clearly attempts to hyperbolize the attacks and change it from a terrorist attack into an Islamic one. The stories and date of the attacks in both quotations (27) and (28) are presented clearly. The past tense is used to make the events clear, factual, and influential. Number game is another strategy used by Trump for interests. Numbers of the victims are mentioned clearly. Trump knows that numbers and statistics are important in making someone sounds more accurate and credible. The killing and wounding of innocent people with their numbers are more affective and influential. In quotation (29), Trump compares presidents Ronald Reagan and Obama in the form of a sport metaphor. President Reagan is a very good player. He always wins and scores goals, while president Obama is a very bad player. He always loses. The discourse of US and THEM is clear in quotation (29). According to Trump, Muslim nations are violating human rights and financing global terrorism. They are bad and negative. In Trump's philosophy, all of these negative people and negative things should be eliminated and at the same time America's greatness will return. This will not be achieved without his leadership. Trump identified himself as a powerful actor that can change all these bad and negative things into good and positive as it can be seen in Table 1 below:

**Table 1:** Trump's racial, divisive, and polarized discourse (US or SELVES and THEM or OTHERS).

US (SELVES)		THEM (OTHERS)	
Christians	Good, positive, innocent, peaceful, targeted, wise, persecuted, and victims	Muslims	Bad, negative, terrorists, violent, peace- haters, spiteful, murderers, vengeful, evil, and enemy
Israel	Ally	Iran	Enemy
Native Americans	Poor, marginalised, stolen rights, deceived, powerless, unimportant, and neglected	Immigrants and Refugees	Ascendant, dominant, important, powerful, dangerous, evil, destructive, and deceptive
Trump	Good, positive, compassionate, sympathetic, wise, equitable, respectful, understandable, reformer	Obama and Clinton	Bad, negative, wrong, reckless, thoughtless, careless, false, incredible, weak, disastrous, rudderless, aimless, senseless, and destructive

## 5 Conclusions

Trumps political speeches are characterized by their hostility and racism towards Muslims and other immigrants in the United States. In the analyzed quotations, I have found that what Trump cares for is the original-native Americans and Christians only.

Trump's anti-Muslim rhetoric might motivate Americans to indulge in violence with the Muslims. In addition to his urgent calls for a ban on Muslims entering the United States, Trump says "Islam hates us" and accuses American Muslims of protecting terrorists. Trump mentions the phrase "Islamic terrorists" and the phrase "radical Islam" many times. Trump is always sympathetic towards the innocent Americans and Christians because of the terrorist acts made by Muslims. This discourse incites Americans to indulge in hate-crime.

Trump deliberately uses the discourse strategies in a more manipulative and persuasive way to achieve political gains against his opponents, especially Muslims, Hillary Clinton and those who support Clinton in the election campaign for the U.S. presidency. Trump always tries to motivate the Native Americans against Muslims, other immigrants, and Obama and Clinton administrations. Trump often exaggerates the way "Islamic terrorists" kill and marginalize innocent Christians in the United States and Europe forgetting about all the heinous crimes that those terrorists, which have nothing to do with Islam, have made to Muslims in Iraq and Syria. He also exaggerates the Obama and Clinton wrong policies deliberately to achieve political goals and win the U.S. presidency.

In most of the quotations, Trump's language is sympathetic and compassionate, full of blames, and negative comparisons (Muslims, immigrants, Obama, and Clinton). Trump always presents himself as sympathetic with the Christians who have been killed and marginalized by "Islamic Terrorists" and neglected by Obama and Clinton wrong administrations. Trump always thinks and speaks about the negative and bad qualities of Muslims, immigrants, and Obama and Clinton wrong policies. He presents the Muslims as haters to others such as Christians, Jewish and "LGTBQ" community. Hate speech, whether it is hard or soft, can raise serious concerns in terms of intolerance and discrimination (Assimakopoulos et al., 2017).

Trump uses the image of racial discrimination clearly in most of the analyzed quotations. The positive and good discourse of US is represented by Trump and the majority of American people. The negative and bad discourse of THEM is represented by immigrants, Muslims, Obama and Clinton administrations. Such racial discrimination can have negative impacts on the peaceful coexistence of not only the American people but also the people of the whole world. Politicians should also avoid using racial and sectarian discourse just because such discourse helps them achieve their personal interests. Politicians should not think about their personal interests as much as they must think about people's interests, needs, and problems. People also should develop their critical literacy and critical language awareness to help them uncover how language can reproduce and promote racism, sectarianism, injustice, and social inequality.

## Declarations

### Author contribution statement

Enas Naji Kadim: Conceived and designed the experiments; Performed the experiments; Analyzed and interpreted the data; Contributed reagents, materials, analysis tools or data; Wrote the paper.

### Funding statement

This research did not receive any specific grant from funding agencies in the public, commercial, or not-for-profit sectors.

### Data availability statement

Data included in article/supplementary material/referenced in article.

### Declaration of interests statement

The authors declare no conflict of interest.

### Additional information

No additional information is available for this paper.

## Appendix

### Quotation 1

Islam hates us. There is a tremendous hatred there. We can't allow people who have this hatred coming into the United States and of people who are not Muslims. It is very hard to define radical Islam from the mainstream practice of the religion, because you don't know who's who.

### Quotation 2

Last December, 14 innocent Americans were gunned down at an office party in San Bernardino, another 22 were injured.

### Quotation 3

In June, 49 Americans were executed at the Pulse Nightclub in Orlando, and another 53 were injured. It was the worst mass shooting in our history, and the worst attack on the LGTBQ community in our history.

### Quotation 4

In January of 2015, a French satirical newspaper, Charlie Hebdo, was attacked for publishing cartoons of the prophet Mohammed. Twelve were killed, including two police officers, and 11 were wounded. Two days later, four were murdered in a Jewish Deli.

### Quotation 5

In March of this year, terrorists detonated a bomb in the Brussels airport, killing 32 and injuring 340.

### Quotation 6

This July, in the South of France, an Islamic terrorist turned his truck into an instrument of mass murder, plowing down and killing 85 men, women and children-and wounding another 308. Among the dead were 2 Americans-a Texas father, and his 11-year-old son.

### Quotation 7

A few weeks ago, in Germany, a refugee armed with an axe wounded five people in a gruesome train truck. Christians driven from their homes and hunted for extermination. ISIS rounding-up what it calls the "nation of the cross" in a campaign of genocide. We cannot let this evil continue.

### Quotation 8

Our foreign policy is a complete and total disaster. No vision, no purpose, no direction, no strategy. President Obama has weakened our military by weakening our economy. He's crippled us with wasteful spending, massive debt, low growth, a huge trade deficit and open borders. I am the only person running for the Presidency who understands this problem and knows how to fix it.

### Quotation 9

We've had a president who dislikes our friends and bows to our enemies. He negotiated a disastrous deal with Iran. Iran cannot be allowed to have a nuclear weapon and, under a Trump administration, will never be allowed to have a nuclear weapon. Israel, our great friend and the one true democracy in the Middle East, has been snubbed and criticized by an administration that lacks moral clarity.

### Quotation 10

Our rivals no longer respect us. When President Obama landed in Cuba on air force one, no leader was there to meet or greet him. Then, amazingly, the same thing happened in Saudi Arabia-it's called no respect.

### Quotation 11

We left Christians subject to intense persecution and even genocide. And we're in a war against radical Islam, but President Obama won't even name the enemy! Hillary Clinton also refuses to say the words "radical Islam," even as she pushes for a massive increase in refugees. After Secretary Clinton's failed intervention in Libya, Islamic terrorists in Benghazi took down our consulate and killed our ambassador and three brave Americans. Then, instead of taking charge that night, Hillary Clinton decided to go home and sleep! Incredible.

### Quotation 12

*We must stop importing extremism through senseless immigration policies*

### Quotation 13

We desire to live peacefully and in friendship with Russia and China. Russia has also seen the horror of Islamic terrorism.

### Quotation 14

With President Obama and Secretary Clinton we've had a reckless, rudderless, and aimless foreign policy-one that has blazed a path of destruction in its wake.

### Quotation 15

Americans must know that we are putting the American people first again. On trade, on immigration, on foreign policy-the jobs, incomes and security of the American worker will always be my first priority. Under a Trump administration, no American citizen will ever again feel that their needs come second to the citizens of foreign countries.

### Quotation 16

When Mexico sends its people, they're not sending their best. They're not sending you. They're not sending you. They're sending people that have lots of problems, and they're bringing those problems with us. They're bringing drugs. They're bringing crime. They're rapists.

### Quotation 17

*I will view the world through the clear lens of American interests. I will be America's* ***greatest*** *defender and* ***most*** *loyal champion. We will not apologize for becoming successful again, but will instead embrace the unique heritage that makes us who we are.*

### Quotation 18

I am your voice!

I have embraced crying mothers who have lost their children because our politicians put their personal agendas before the national good. I am going to bring our jobs back to Ohio and Pennsylvania and New York and Michigan and all of America – and I am not going to let companies move to other countries, firing their employees along the way, without consequences. Not going to happen anymore.

### Quotation 19

It is time for a change. We need a change. A real change, not an Obama change. We don't need an Obama change. We have had enough. It is time to change a rigged political system that works only for the insiders, and replace it with a government that serves the people.

### Quotation 20

*She is a****dangerous****liar who has disregarded the lives of Americans, and who has put all of us at great risk while* supporting *economic policies that have destroyed our economy. We have a long way to go.*

### Quotation 21

*So, listen, so we have people coming to our country that should not be coming to our country. We have* ***thousands*** *and* ***thousands*** *of people from certain terrorist states, from certain parts of the world that we are allowing to come into our country.* ***Most*** *of the politicians,* ***almost*** *all of them don't even know where they are, where they are being put, they know nothing about these people. This could be the great Trojan Horse of all time. And Hillary wants* ***thousands*** *more to come in over and above Obama. Right? The Boston bombers" --* ***remember those horrible, disgusting*** *people? "The Boston bombers arrived through the political asylum process.*

### Quotation 22

The Moroccan national who came to the United States on a student visa was arrested for plotting to blow up a university in a federal courthouse. Great. A Uzbek refugee living in Idaho was arrested and charged with providing support to a terrorist organization in the form of teaching terror recruits how to build massive and very dangerous bombs.

### Quotation 23

An immigrant from Syria who later applied for and received U.S. citizenship was accused by federal prosecutors of planning to go to a military base in Texas and kill three or four American soldiers, execution style if possible -- more than that, if at all possible. A college student who immigrated from Somalia who later applied and received U.S. citizenship, attempted to blow up a Christmas tree lighting ceremony in Oregon.

### Quotation 24

An Iraqi immigrant who later applied for and received U.S. citizenship was arrested for lying to federal agents about pledging allegiance to ISIS, his travels to Syria, and how many Americans he wanted to kill. Two immigrants from Pakistan who later applied for and received U.S. citizenship were sentenced to decades long prison sentences for plotting to detonate a bomb -- and a very big bomb, in the middle of New York City.

### Quotation 25

An immigrant from Yemen who later applied for and received U.S. citizenship was arrested for trying to join ISIS. He was also charged with attempting to illegally buy firearms to try to shoot as many American military personnel as possible.

### Quotation 26

An immigrant from Afghanistan who later applied for and received U.S. citizenship, and a legal permanent resident from the Philippines were convicted for plotting to join Al Qaida and the Taliban in order to kill as many Americans as possible -- as many as possible.

### Quotation 27

In Europe--thank you--in Europe we have seen the same carnage and bloodshed afflicted upon our closest allies. In January of 2015, a French satirical newspaper, Charlie Hebdo, was attacked for publishing cartoons of the prophet Mohammed. Twelve were killed, including two police officers, and 11 were wounded. Two days later, four were murdered in a Jewish delicatessen. In November of 2015 terrorist went on a shooting rampage in Paris that slaughtered 130 people, and wounded another 368 people, some in very, very, very bad shape today.

### Quotation 28

France is suffering gravely. And the tourism industry is being massively affected in the most negative way. In March of this year terrorists detonated a bomb in Brussels airport killing 32 and injuring 340 people. This July in the South of France an Islamic terrorist turned his truck into an instrument of mass murder, plowing down and killing 85 men, women and children and wounding 308 people.

### Quotation 29

In winning the Cold War, President Ronald Reagan repeatedly touted the superiority of freedom over communism and called the USSR the Evil Empire. Yet when President Obama delivered his address in Cairo, no such moral courage could be found or would be found. Instead of condemning the oppression of women and gays in many Muslim nations, and the systematic violations of human rights, or the financing of global terrorism, President Obama tried to draw an equivalency between our human rights record--and remember this, our human rights--and theirs.

### Quotation 30

I am the change agent. I am the change agent. And I will work for you and I will work for no one else. I will work for you. I will never lie to you. I will never put any other interests before you and I will never, ever stop fighting for you.
